# Case report: Joint diagnosis and treatment of intrathoracic gastric duplication with a gastric communication in a child by laparoscopy and gastroscopy

**DOI:** 10.3389/fped.2023.1143741

**Published:** 2023-03-15

**Authors:** Huashan Zhao, Yunpeng Zhai, Rui Guo, Hongxiu Xu, Longfei Lv, Shisong Zhang

**Affiliations:** ^1^ Department of Thoracic and Oncological Surgery, Children’s Hospital Affiliated to Shandong University, Jinan, China; ^2^ Department of Thoracic and Oncological Surgery, Jinan Children’s Hospital, Jinan, China

**Keywords:** laparoscopy, gastroscopy, diagnosis, treatment, child, intrathoracic gastric duplication

## Abstract

Intrathoracic gastric duplication has rarely been reported. A 5-year-old child with gastric duplication located in the left thorax was diagnosed and treated successfully using laparoscopy combined with gastroscopy. Preoperative computed tomography, upper gastrointestinal contrast study, ultrasound, and other imaging methods were insufficient for accurate diagnosis in this case. Laparoscopy combined with gastroscopy is more suitable for the diagnosis and treatment of gastric duplication.

## 1. Introduction

Gastric duplication is a rare congenital malformation of the digestive tract that originates from embryonic development; it is even rarer when it occurs in the thoracic cavity. Several cases of gastric duplication have been reported in the abdominal cavity, but fewer are noted in the thoracic cavity. We report a case of gastric duplication in the left thoracic cavity, connected to the stomach. Gastric duplicates account for 2%–8% of all gastrointestinal duplicates, with an incidence of approximately 17 case/1 million (1, 2). Gastric duplication usually occurs at the greater curvature of the stomach (3). Diagnosis and treatment of gastric duplication were successfully accomplished using laparoscopy combined with gastroscopy in our case.

## 2. Case description

The patient was a 5-year-old boy. Seven days before this admission, he was admitted to the hospital due to pneumonia and was incidentally found to have a mass occupying the left mediastinum during computed tomography (CT) examination. This was initially thought to be either an esophageal foramen hernia or pulmonary isolation syndrome. He had no chest tightness, pain, dyspnea, cough, hemoptysis, or other clinical symptoms. Physical examination demonstrated a normal thoracic cavity, regular respiratory rhythm, symmetrical respiratory movements, and normal intercostal spaces. Respiratory sounds were coarse in both lungs, no rales were heard, and no prolonged expiratory phase was observed. Laboratory examination revealed normal routine blood markers, including liver and kidney function, electrolytes, coagulation indexes, and amylase levels. Preoperative upper gastrointestinal tract contrast study showed an irregular posterior esophageal lumen shadow and an esophagogenic cyst.

**Figure 1:**
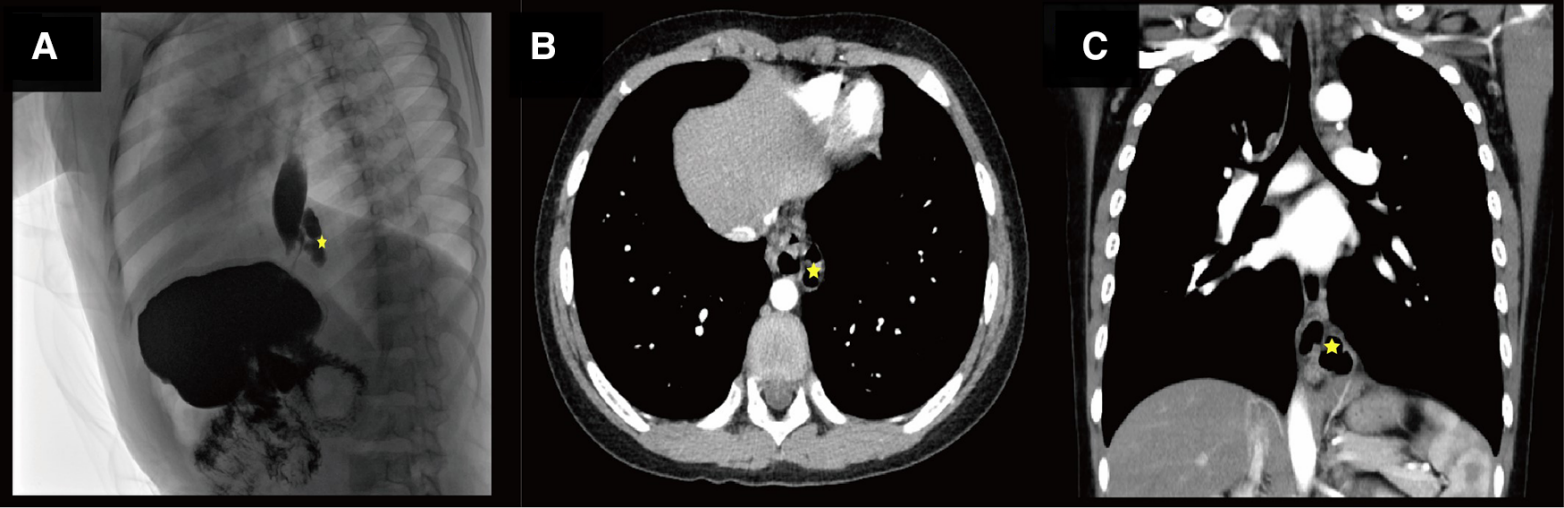
(**A**) Shows the radiological investigation of the child. Star indicates an irregular lumen shadow behind the lower esophageal segment on the lateral view of the upper gastrointestinal contrast study before the operation. (**B**) Shows the radiological investigation of the child. The star indicates an irregular lumen in the left posterior mediastinum of the contrast-enhanced computed tomography (CT) before the operation. (**C**) Shows the radiological investigation of the child. The star shows an irregular lumen shadow adjacent to the lower esophagus on coronal contrast-enhanced CT before the operation.

As seen in Figure 1A, enhanced CT displayed an irregular cystic air cavity shadow with a slightly thickened cyst wall and a local gas–liquid plane at the upper margin of T9–11 on the left posterior mediastinum. The lesion was 30.4 × 17.3 × 30.0 mm, and the cyst wall was further visualized using enhanced scanning. The esophagus moved anteriorly with pressure, blurring the boundary adjacent to the left inferior phrenic artery branch; the adjacent lung tissue was also changed under pressure (Figures 1B,C).

**Figure 2:**
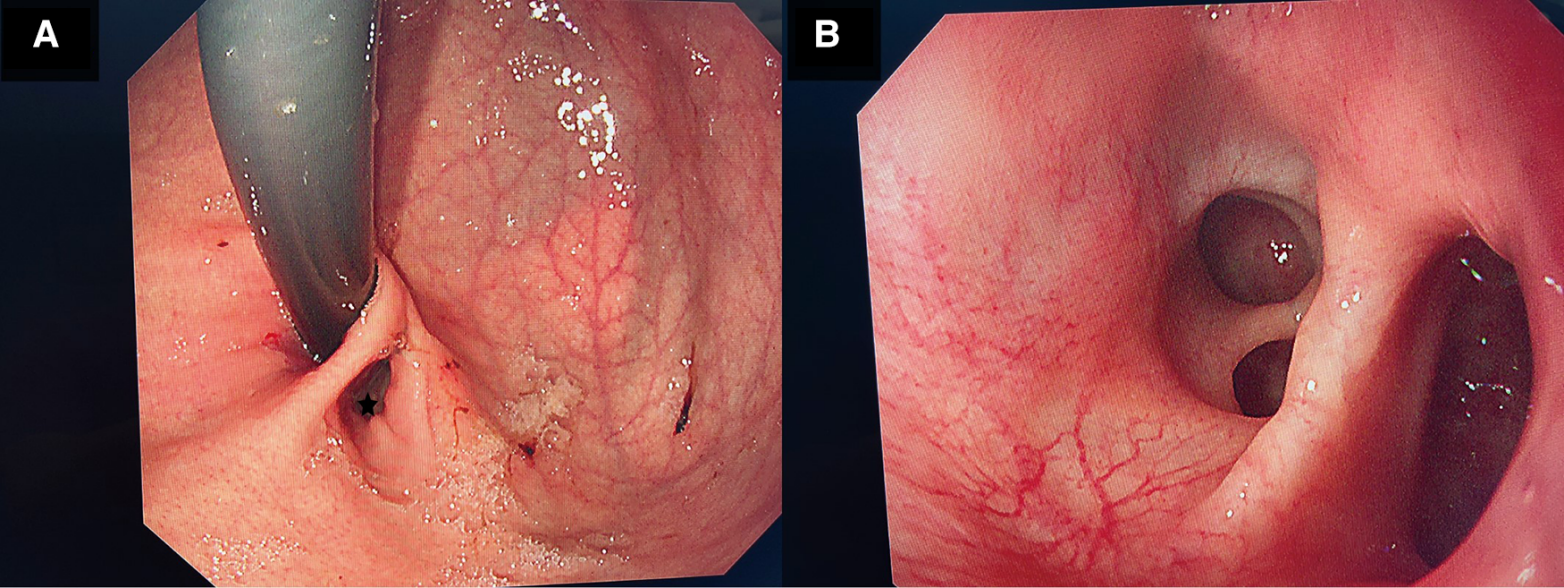
(**A**) Shows the gastroscopy of the child. There is an abnormal opening (gastric duplication opening) in the gastric fundus beside the cardia during pentagram gastroscopy. (**B**) Shows the gastroscopy of the child. Multiple open cysts are seen after the gastroscope is moved along the opening of the repeated malformation of the stomach.

**Figure 3:**
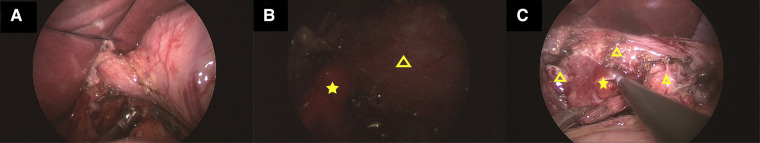
(**A**) Shows the radiological investigation of the child during laparoscopy: the gastric fundus and its adjacent digestive tract surface are smooth and normal, and no prominent lesions are observed. (**B**) Shows the radiological investigation of the child during laparoscopic surgery. The red bright light (starred) indicates the positioning of the gastroscope, and the triangle is the position of the stomach. (**C**) Shows the radiological investigation of the child during laparoscopic surgery. The star is the lesion located in the smooth muscle layer of the digestive tract, and the triangle is the smooth muscle layer of the digestive tract outside the lesion.

**Figure 4:**
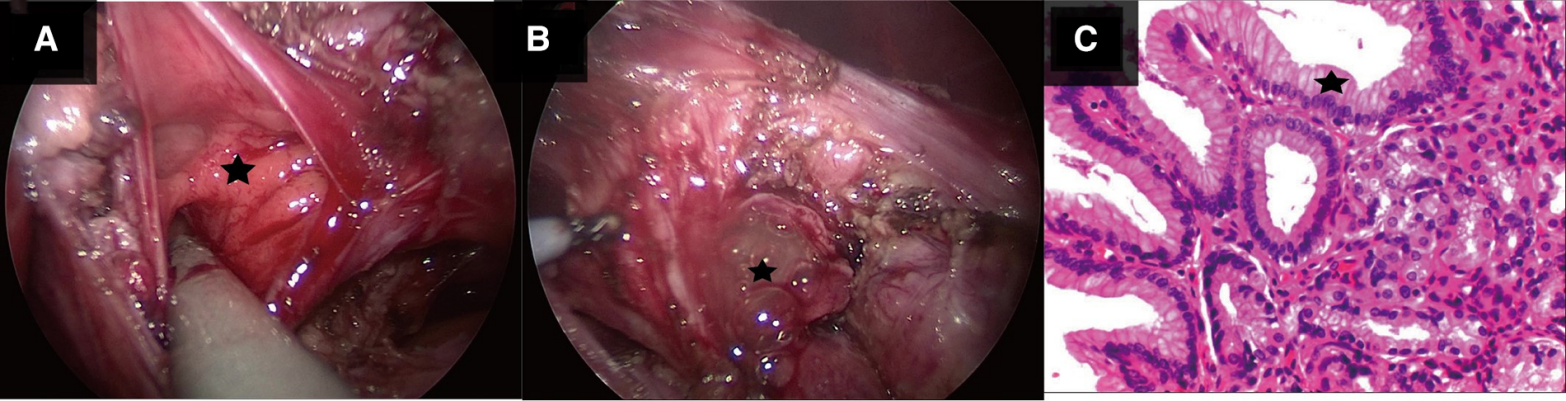
(**A**) Shows the child during laparoscopic surgery. The star shows that there are multiple openings of interconnecting sacs in laparoscopic repetitive malformation. (**B**) Shows the child during laparoscopic surgery. The star shows gelatinous cystic fluid outflow in laparoscopic repetitive malformation. (**C**) Shows the pathological image of the child. The star shows the gastric mucosa indicated in the pathological section stained with hematoxylin and eosin stain (magnification ×400).

After preoperative discussion, the possibility of an esophageal cyst was considered high, but other digestive tract malformations could not be ruled out. The presence of lesions and the parents' concerns about the progression of the disease spurred the decision to perform a surgical exploration. After successful anesthesia. Gastroscopy revealed an abnormal opening in the gastric fundus paracardia, as shown in Figure 2A. A multilocular cyst was found along the opening. No ulcers or food residues were found in the area, as shown in Figure 2B. The gastroscopy concluded that the lesion opened in the gastric fundus and extended upward into the thoracic cavity. A curved incision was made at the lower edge of the umbilical cord, about 1.0 cm long. A 10-mmTrocar was implanted to establish a CO2 pneumoperitoneum with a pressure of 6 mmHg and a flow of 2 L/min. A small 5-mm incision was made from the left middle and upper abdomen and the right middle and upper abdomen, respectively, and a 5-mmTrocar was inserted into each puncture under direct vision. It was found that the digestive tract surface of the gastric fundus and its vicinity were smooth and normal, and no prominent lesions were observed, as shown in Figure 3A. Intraoperative positioning was combined with gastroscopy, as shown in Figure 3B. The lesion was found to be adjacent to the mucosa of the stomach and esophagus after the incision of the posterior part of the stomach and the serous and smooth layer of the digestive tract. The lesion was located in the smooth muscle layer of the digestive tract. It extended along the smooth muscle layer of the esophagus into the chest, as shown in Figures 3C, 4A,B. The anterior wall of the esophageal hiatus was reflected to expose the esophageal hiatus. The esophageal muscle layer was opened to expose the lesion. Due to the depth of the lesion, the lesion mucosa was dissected. After no residual lesions were found, the gastric wall was repaired, and the gastric fundus, esophageal muscle layer, and serous layer were repaired. Gastroscopy showed good repair of the esophagus and gastric fundus, with no anastomotic fistula or remaining leakage. The esophageal hiatus was repaired and an indwelling abdominal drainage tube was left in place. The incision was sutured layer by layer and the operation was completed.

**Figure 5:**
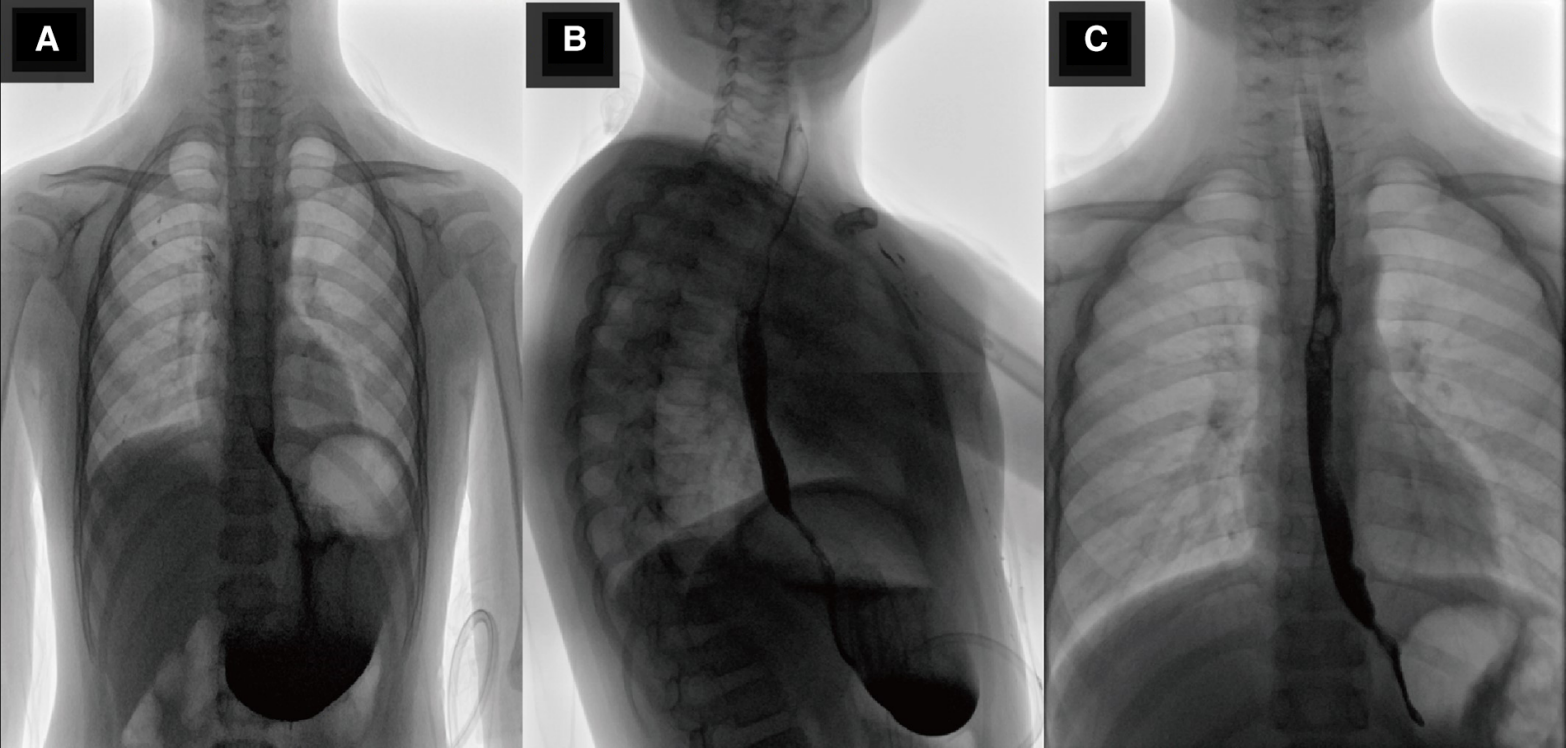
(**A,B**) Show the radiological investigation of the child: positive and lateral upper gastrointestinal radiographs reviewed 10 days after surgery, showing good recovery. (**C**) Shows the radiological investigation of the child: re-examination of the upper gastrointestinal tract contrast study 2 months after surgery, showing good recovery.

After the surgery, antibiotics, fluid replacement, and other supportive treatments were administered. Diet was resumed 7 days after the operation, and the abdominal drainage tube was removed 10 days after the operation. Most of the submitted pathological samples were lined with gastric mucosa, and a small part of the lining epithelium was shed, with slight hyperplasia of granulation and fibrous tissues (Figure 4C). Upper gastrointestinal tract contrast study was reviewed 10 days after surgery (Figures 5A,B), and the abdominal color ultrasound demonstrated excellent recovery with no abnormalities. No obvious discomfort was reported during the telephone interviews 1 and 7 months after discharge. Upper gastrointestinal angiography was reviewed 2 months after surgery (Figure 5C), with good recovery observed.

## 3. Discussion

Gastrointestinal duplication is a rare congenital malformation that can occur anywhere in the gastrointestinal tract (from the mouth to the anus). Almost half of the malformations occur in the small intestine, especially in the ileum, and rarely in other sections. Gastric duplicates account for 2%–8% of all gastrointestinal duplicates (1), with an incidence of approximately 17 cases in 1 million (2). Gastric duplication usually occurs at the greater curvature of the stomach. Gastric duplication in the left thoracic cavity, as in our case, is extremely rare.

The manifestations of gastric duplication in children of different ages vary. Patients may experience abdominal pain, vomiting, bloody stools, difficulty eating, or other non-specific gastrointestinal symptoms (4–6). Gastric duplication can be divided into tube, cyst, and endogastric types (7). At present, the diagnostic methods for gastric duplication include ultrasound, CT, upper gastrointestinal contrast studies, isotope imaging, and gastroscopy (1). Preoperative diagnosis of gastric duplication is difficult, and it is sometimes easy to misdiagnose cystic masses based on CT and ultrasound examination results alone (8, 9). In this case, the preoperative diagnosis was very difficult, and the final diagnosis was achieved using laparoscopic, combined with gastroscopic and pathological approaches.

Some studies have found that cases of gastric duplication may undergo malignant transformation (9–11). For this reason, surgical resection is the treatment of choice. Surgical methods include gastric duplication cyst excision, partial gastric resection and gastric repair, and endoscopic mucosal excision (12–15); the approach should be selected according to patient characteristics. Preoperative exams failed to lead to a definitive diagnosis. To determine the diagnosis and treatment strategy, gastroscopy was firstly performed; and gastric duplication cyst was diagnosed. Intraoperative gastroscopy-assisted laparoscopy was effective to search for gastric duplication lesions.

Gastric duplication deformity, which can communicate with the stomach cavity, is rare (16). Rowling (7) put forward the following pathological diagnostic criteria of gastric duplication: the focal capsule wall surrounds the smooth muscle layer and covers the mucous membrane of the digestive tract. The foci are attached to the gastric wall and share the blood supply. Gastric diverticula are cystoid bulges of the stomach wall outside the stomach wall; they are divided into congenital and acquired types, such as congenital muscular dysplasia and acquired muscular deficiency (17, 18). Gastrointestinal contrast study of the gastric diverticulum shows isolated pocket-like, spherical, or hemispherical filling shapes protruding from the stomach cavity (19). In our case, association with the stomach could easily be misinterpreted as gastric diverticulum.

In this case, the lesion shared the muscle layer with the gastric digestive tract and was located in the smooth muscle layer, and pathological examination showed gastric mucosa. The above are in line with the pathological diagnostic criteria of gastric duplication. No protruding masses were found on the surface of the stomach during laparoscopy, which is inconsistent with the bulge of a gastric diverticulum outside the stomach wall. A multilocular traffic cyst was found; hence, our case is multi-chamber gastric duplication with gastric contact extending into the thoracic cavity.

Gastric duplication with an intrathoracic connection to the stomach is rare and difficult to diagnose. The combination of laparoscopy and gastroscopy in the treatment of intrathoracic and gastric-connected gastric duplicates is of great clinical significance. The case that we encountered in our surgical practice is extremely unique, and no other similar cases could be found in the published literature. This study can raise awareness of the differential diagnosis and treatment among clinicians who might encounter similar cases in the future. Finally, the patient had a good prognosis and recovered well after the review.

## Data Availability

The original contributions presented in the study are included in the article/Supplementary Material, further inquiries can be directed to the corresponding author.
